# Research capacity strengthening for sexual and reproductive health: a case study from Latin America

**DOI:** 10.1186/s12978-016-0222-0

**Published:** 2017-03-07

**Authors:** Rita Kabra, Marco Castillo, Mercedes Melián, Moazzam Ali, Lale Say, A. Metin Gulmezoglu

**Affiliations:** 1 Department of Reproductive Health and Research including UNDP/UNFPA/UNICEF/WHO/World Bank Special Programme of Research, Development and Research Training in Human Reproduction, World Health Organisation, Avenue Appia 20, Geneva, 1211 Switzerland; 2 Paraguayan Center for Population Studies (CEPEP), Asunción, Paraguay; 3 22 chemin Henry Wissner, Grand Lancy, Geneva, 1212 Switzerland

**Keywords:** Research capacity strengthening, Institutional development, Sexual and reproductive health, Low- and middle-income countries (LMICs), Grants, Policy, Practices

## Abstract

An essential, but often overlooked part of health promotion and development support to achieve self-sufficiency in developing countries is the concomitant need to build and strengthen research capacity. This is even more challenging and critical in the area of sexual and reproductive health because of diverse interplay of socio cultural, religious, economic factors in relation to reproductive health.

This paper presents the case study of HRP’s efforts to build research capacity in Latin America by studying and analyzing the 5-year history of institutional development support to an institution in Paraguay. In reviewing the efforts, we identify the strengths in the approaches used by HRP, the challenges and outcomes of the process and we present recommendations for future efforts to strengthen research capacity to improve sexual and reproductive health. The authors call for greater support from and collaborative efforts of developmental partners and governments to strengthen research capacity in low and middle-income countries to improve sexual and reproductive health.

**Electronic supplementary material:**

The online version of this article (doi:10.1186/s12978-016-0222-0) contains supplementary material, which is available to authorized users.

## Multilingual abstracts

Please see Additional file 1 for translations of the abstract into the three official working languages of the United Nations.

## Plain English summary

This paper presents the case study of WHO-HRP’s efforts to build research capacity in Latin America by studying and analyzing the 5-year history of institutional development support to the Paraguayan Center for Population Studies (CEPEP) in Paraguay.

We explain the strengths in the approaches used by HRP, the challenges and outcomes of the process and present recommendations for future efforts to strengthen research capacity to improve sexual and reproductive health.

Greater support and collaborative efforts of developmental partners and governments is required to strengthen research capacity in low and middle-income countries to improve sexual and reproductive health.

## Background

The UNDP/UNFPA/UNICEF/WHO/World Bank Special programme of Research, Development and Research Training in Human Reproduction (HRP) is widely known and is the only programme within the United Nations system with a global mandate to lead research and to conduct research capacity strengthening efforts in the field of sexual and reproductive health (SRH) and rights [1]. HRP conducts, supports and coordinates research on a global scale, synthesizes research through systematic reviews of literature, builds research capacity in low-income countries and develops dissemination tools to make efficient use of ever-increasing research information. Since HRP’s inception in 1972, it has provided long-term support to 103 institutions in 55 countries, to develop their research capacity, address priority needs and build national self-reliance in research on all aspects of human reproduction [1].

The World Health Report 2013 referred to research capacity strengthening as the abilities of individuals, institutions and networks, nationally and internationally, to undertake and disseminate research findings of the highest quality [2]. Strengthening research capacity in low-and middle-income countries (LMICs) is one of the most powerful, cost effective and sustainable means of advancing health, health care and development [3].

The explicit modalities of Research Capacity Strengthening (RCS)/HRP have the comparative advantage of providing support to institutions in LMICs though a comprehensive technical support package, which includes the development and strengthening of the necessary technical infrastructure to support the conduct of research [1]. It includes short and long-term training of staff in areas identified by the local institutions as critical for human resource development.

This paper presents a case study of HRP’s efforts to build research capacity in Latin America by studying and analyzing the 5-year history of institutional development support to one institution in Paraguay, which had no previous experience in global research. In reviewing the efforts, we identify the strengths in the approaches used by HRP to strengthen research capacity, the challenges, and outcomes of the process and present recommendations for future efforts to strengthen research capacity to improve SRH.

## Study objectives and methods

The objective of this review was to identify the outcomes and impact of HRP’s efforts and to learn from these past efforts and contribute to the HRP’s future RCS programme by analyzing the 5 year period of research capacity strengthening at one institute in Paraguay. Data was collected through contacts with the institutions staff during site visit, analysis of reports from the institutes and personal communications with staffs from mentor and mentee institutions.

### Study setting

#### Paraguay demographics

Paraguay is a landlocked country in the middle of South America with approximately 7 million inhabitants. The country is among the poorest in the region and has a relatively young democracy. Democracy was restored after a 35-year dictatorial regime, which left several areas of social development such as education, social service, and health care underserved. Paraguay’s most recent 5 years National Reproductive Health Plan (2014–2018) [4] outlines seven priorities for the implementation of public policies, actions and research, namely: safe maternal and neonatal health, access to family planning, prevention of sexually transmitted infections, prevention of breast cancer in women and genital cancer in men, integral attention of women at the menopausal and post-menopause as well as to reproductive dysfunction, prevention and attention of victims of gender based violence, and efficient management and monitoring of the plan [4].^.^

### The Paraguayan Center for Population Studies

The Paraguayan Center for Population Studies (CEPEP) www.cepep.org.py [5] is a non-governmental organization founded on March 1966. Since inception CEPEP has played a pioneering role in promoting SRH in the country by offering reproductive health care services in poor neighborhoods of Asuncion (the capital) and the countryside. In 1975, the Ministry of Public Health started to offer family planning services but this decision was revoked in 1979 [6]. In addition, during the period 1979–1988 the government suspended provision of contraceptives. CEPEP was the only institution that continued to offer SRH services emphasizing contraception, adolescents and reproductive rights through their 18 clinics scattered around the country during this decade. Today CEPEP runs four reproductive health clinics located in Asuncion, San Lorenzo (suburb area of Asuncion), Ciudad del Este (second largest city, east of Asuncion, at the Brazilian border), and Encarnacion (third largest city, south of Asuncion, at the Argentinean border).

### Selection of CEPEP for strengthening research capacity through HRP-LID Grant

To strengthen human and material resources for reproductive health research in Paraguay, HRP staff visited Paraguay and had discussions with the Ministry of Health in 2007. Following a screening process based on the eligibility criteria [7] (see Table 1) and in collaboration with the Regional Advisory Panel (RAP-HRP Governing body) for the Americas, the Paraguay-Ministry of Health and WHO country office; CEPEP was selected to receive first–Long-term institutional development (LID) [8] grant in 2008. The LID grant provided funding to the center for the implementation of well-defined research projects; research training, organization of short group learning activities; purchase of computer equipment and software, including establishment of information technology facilities; data processing and library resources.

**Table 1:** Eligibility criteria for LID grant [8]

• The centre should be a research unit focused on reproductive health (RH) and be part of a university, government structure or a non-governmental organization.
• The centre, should demonstrate the potential for becoming a viable research entity, responsive to national RH needs.
• LID grant applications should have the support of national and immediate authorities.
• Applications ashould preferably be from centres in Least Developed Countries (LDC).
• The centre should provide evidence that it has the necessary financial support structures and essential administrative leadership to implement, the grant proposal
• The centre should have the potential to apply the research findings in RH care and prevention by having established or establishing firm links with appropriate policy-makers and stakeholders as well as SRH programmes and services.
• Recipients of two consecutive cycles of LID grants are not eligible.
• Prospective centres should have, or be willing to establish, acceptable scientific and ethical review mechanisms for research.

Within a program of South-South collaboration devised by HRP/RCS to develop the expertise of emerging centres for research, the Centro de Estudios de Poblacion www.cenep.org.ar (CENEP) [9] in Argentina was given a research project mentoring (RPM) grant to mentor CEPEP in acquiring the technical skills to conduct high quality scientific research in SRH.

### Outcome and potential impact following HRP support

Training and research: During the first 5 years of the LID grant (2009–2014), based on gaps identified by the institution, training support was provided on data analysis (quantitative/qualitative), multivariate analysis, scientific writing, dissemination of research results, and library management. The training programme included a hands-on component using data from CEPEP’s databases and current research. Another form of training was a result of the continuous advice from CENEP on developing the research protocol (budgeting the activities, ethical aspects in conducting research, bibliography search), data analysis and publication of research findings (learning by doing). A mentor- mentee relationship developed between the two institutions with staff visiting each other’s institution to work together on developing and implementing research protocols.

Following the training, technical support from HRP and continuous mentoring by CENEP, CEPEP conducted several research projects. In 2011 they conducted the study *“Women at risk: Detection of target groups of women at higher risk of infection by HIV, unplanned fertility, fetal loss and violence victimization in Paraguay”* and published the results [10–12]. Based on these findings, the institution designed another study on “*Intimate partner violence and reproductive coercion against women”* that aimed to describe the individual and societal mechanisms and circumstances around violence against women in Paraguay. The results were presented in the Regional Seminar on Intimate Partner Violence held in Asuncion in 2015 with the support of WHO http://www.cepep.org.py/Seminario2015/Ponencia.html). A manuscript is currently under review for publication. In 2015, the researchers began elaborating another research study–*Attitudes, opinions and experiences of partner violence from the perspective of men in Asuncion.* HRP staff worked with CEPEP in developing the study protocol. The study was approved by HRP scientific committee and is currently under WHO Ethical review. It will be implemented in the last quarter of 2015.

Knowledge resource management: An important contribution of the HRP/LID grant was the reorganization and expansion of CEPEP’s library. In 2009, an expert librarian from CENEP conducted a capacity building workshop on “Electronic Resources Management”. Nineteen librarians from 14 different libraries in Paraguay attended the course, including the librarian from the School of Medicine of the National University of Asuncion, from the General National Direction of Statistics, and the Central library of the University of Asuncion. In the post-course evaluation, all participants remarked, *“that it was the first time they had the opportunity to be trained in such advanced tools for library management”*. Through the LID grant, library was able to purchase and enhance the collection of reference books and documents. Approximately 104 books in the discipline of social sciences, research methodology, SRH, psychology, and epidemiology were purchased and subscriptions to local journals were made. The training course for librarians, helped to create linkages within this small community of librarians in Paraguay and to share knowledge not otherwise easily accessible in the country. HRP support to the library not only enhanced knowledge generation for CEPEP’s researchers but it served as a resource tool for the general public of Paraguay as well as neighboring countries, thus creating a research milieu in the country.

Dissemination of research findings: One of the objectives of HRP/RCS programme is to support researchers from LMICs to disseminate their research findings. Before CEPEP became a LID grantee, CEPEP had published results of SRH surveys, policy briefs, summaries of research results in monograph form. Following the training on scientific writing and dissemination of research results, there began a shift towards publishing in national and international scientific peer reviewed–journals, developing policy briefs, participating and organizing congresses and seminars. Research results were published on intimate partner violence, mental health, fertility decline [13], and trends of caesarean delivery [14]. Evaluation of the National Reproductive Health Plan 2003–2008 [15] and data on reproductive health of adolescents and young women [16] were published. A series of policy briefs [17] were produced to disseminate research findings to authorities such as Ministry of Health, Ministry of Women Affaires, other stakeholders such as international partners like UNFPA, UNDP, PAHO/WHO, academic sector, and NGO’s. A total of seven articles on research results were published in peer-reviewed journals (three of them had CEPEP’s researchers as lead authors, and in four as coauthors).

Advocacy and influencing policies: Dissemination of research findings was not only limited to publications. Following collaboration and support from CENEP and HRP, CEPEP staff started participating and organizing national and international seminars. In 2010, CEPEP presented the research findings for the first time outside Paraguay at the Fourth Latin American Congress of Population Studies, Havana, and then at the Twenty-First meeting of the Latin-American Association of Researchers in Human Reproduction in Panama in 2011. In 2012, and 2015 CEPEP took the lead and organized the regional seminars on *Violence among adolescents and youth* [11], and *Gender roles and intimate partner viole*nce in Asunción, Paraguay (supported by HRP/WHO).

By organizing advocacy seminars, CEPEP contributed in creating a scientific environment in Paraguay, increased the visibility of the institute, and presented research findings to different stakeholders. This process, together with CEPEP’s previous work is increasingly contributing in creating a milieu where research results are accepted as important inputs for projects and programs, resulting in more research informed policies and practice. Staff from CEPEP now sits on national SRH research policy planning bodies, thereby influencing the development of reproductive health programmes and the National agenda in their countries. The three latest National Reproductive Health Plans have all been evaluated by and developed in consultation with CEPEP.

Networking and Visibility: CEPEP had very little international exposure and experience in conducting research before 2005. Following the LID grant and association with CENEP they became members of the Latin American Population Association–ALAP, the Latin America Association of Researchers in Human Reproduction–ALIRH and international organizations like the International Union for the Scientific Study of Population (IUSSP). HRP support helped in increasing the credibility, visibility, linkages and confidence of the researchers in CEPEP. The HRP Alliance formed in 2014 aims to link emerging research centres in the region and globally to further enhance regional networking and visibility of research centres.

Sustainability of the institution: Success of RCS programme could be assessed by the extent to which an institution can sustain itself after the grant is finished. The achievement of sustainability is evidenced in recent years by the number of projects and seminars receiving financial support from sources other than HRP. CEPEP is moving in the right direction. Recently they were awarded two grants to conduct research to estimate the magnitude of abortion in Paraguay for the first time and to further develop research on intimate partner violence in cities other than the capital. These will be implemented with the financial support of Paraguay’s first national program to develop scientific research called PROCIENCIA [18], dependent on the National Council for Science and Technology. From sustainability perspective, such activities and initiatives are important to consolidate the leadership role and the visibility of the institution so that it continues to play a key role at national and even at regional level in generating and implementing evidence based knowledge and be part of the regional network in sexual and reproductive health.

## Conclusions

In the case study described above, it must be noted that HRP’s RCS programme has played a key role in creating a research milieu in the country. Before HRP’s input to Paraguay, there were few or no institutions conducting research in reproductive health, research was mainly descriptive in nature most of the publications took the form of reports, newspaper articles; publications by CEPEP researchers were mainly in Spanish, and in local journals.

The strategy of combining the LID and RPM grants served as the major facilitating factors for the contributions and accomplishment of this institute over these years. The mentorship and continued support provided by CENEP and HRP resulted in generation of high quality research, participation and contributions in seminars, dissemination of research findings in English language peer reviewed journals, influencing the national agenda, and practices in the region leading to increased CEPEPs networking, visibility and credibility.

Short-term training in research methodology, data analysis, computing facilities and linkages with other institutions and the library support improved access to scientific literature and created a scientific frame of mind contributing significantly to the research output of the institution and made a significant difference in strengthening research capacity in the country.

To advance research on SRH, researchers in LMICs need continuous and long-term support to undertake research based on national needs and priorities and to disseminate research findings to the decision makers so as to generate evidence based policies and programmes relevant to address sexual and reproductive health of the population. Networking and linking the emerging institutions with mature institutions in the region and with ministries of health promotes sustainability and research skills in the institution.

Though this is an example from Latin America, this case study clearly demonstrates that countries in other regions can similarly benefit from HRP’s strategy of research capacity strengthening like Paraguay. The authors recommend that similar modalities and strategies of linking LID and RPM grants (within the region) be used in other countries/regions and call for greater support from development partners and governments for strengthening research capacity to improve SRH.

### Key recommendations based on lessons learnt

1. Greater support is needed from developmental partners and governments to strengthen research capacity in LMICs to improve SRH.
2. Long term support to institutions to attain a certain degree of capacity to conduct quality research in sexual and reproductive health and to sustain the gains made is critical.
3. Increase collaboration between mature and emerging research centres for further strengthening the research environment.
4. Networking and visibility of local institutions will help garner support, increasing recognition and leading to sustainability.

## Additional file

Additional file 1: Multilingual abstracts in the three official working languages of the United Nations. (DOC 27 kb)

## Abbreviations

ALAP: Latin American Population Association
ALIRH: Latin America association of researchers in human reproduction
CENEP: Centre de estudios de poblacion
CEPEP: Paraguayan Center for Population Studies
HRP: The UNDP/UNFPA/UNICEF/WHO/World Bank Special programme of Research, Development and Research Training in Human Reproduction
LID: Long-term institutional development
LMIC: Low and middle income countries
RCS: Research capacity strengthening
RH: Reproductive health
RPM: Research project mentoring
SRH: Sexual and reproductive health
