# Supplementary material for: Research capacity strengthening for sexual and reproductive health: a case study from Latin America
Source: Reprod Health. 2017 Mar 7;14:35. doi: 10.1186/s12978-016-0222-0 (PMC5339979; doi:10.1186/s12978-016-0222-0)
Supplement: Additional file 1: — Multilingual abstracts in the three official working languages of the United Nations. (DOC 27 kb) [file 12978_2016_222_MOESM1_ESM.doc]

Translation of the abstract into the three official working langauges of the United Nations

**Abstract non English language**

Spanish

Una parte importante en la promoción y desarrollo de autosuficiencia en salud en los países en desarrollo es la necesidad de construir y fortalecer la capacidad de investigación. Esta necesidad es aún más desafiante y crucial en el área de la salud sexual y reproductiva debido a la diversidad de factores socioculturales, religiosos y económicos que interactúan sobre la salud reproductiva.

Este artículo presenta un caso de estudio sobre los esfuerzos de HRP para construir capacidad de investigación en América Latina analizando la historia de cinco años de apoyo al fortalecimiento institucional de una organización en Paraguay. Al revisar el proceso, identificamos las fortalezas de los abordajes usados por HRP, los desafíos así como los resultados más resaltantes. Las y los autores instan a un mayor apoyo y esfuerzos colaborativos para fortalecer la capacidad en investigación en países de bajos y medianos ingresos a fin de contribuir a mejorar la salud sexual y reproductiva.

Palabras clave: Fortalecimiento de capacidades de investigación, fortalecimiento institucional, salud sexual y reproductiva, países de bajos y medianos ingresos, subvenciones, políticas, prácticas.

Abstract Portugese

Resumo
A necessidade de construir e fortalecer a capacidade de pesquisa é uma faceta importante na promoção e desenvolvimento da autossuficiência, em matéria de saúde, dos países emergentes. A mesma necessidade é ainda mais desafiante e crucial na área da saúde sexual e reprodutiva; devido à diversidade de fatores socioculturais, religiosos e econômicos que interagem sobre a saúde reprodutiva.

Este artigo apresenta um estudo de caso sobre os esforços do HRP para construir a
capacidade de pesquisa na América Latina, analisando a história de cinco anos de apoio para o
fortalecimento institucional de uma organização no Paraguai. No processo de avaliação,
identificamos os pontos fortes das abordagens utilizadas pelo HRP, os desafios mesmo que os
resultados mais destacados. Os autores exortam um maior apoio e esforços de colaboração
para reforçar a capacidade de pesquisa nos países de baixa e média renda a fim de contribuir no
melhoramento da saúde sexual e reprodutiva.

Palavras-chave: Fortalecimento de capacidades de pesquisa, Fortalecimento institucional,
Saúde sexual e reprodutiva, Países de baixa e média renda, Subvenções, Políticas, Práticas.

Abstract English

An essential, but often overlooked part of health promotion and development support to achieve self-sufficiency in developing countries is the concomitant need to build and strengthen research capacity. This is even more challenging and critical in the area of sexual and reproductive health because of diverse interplay of socio cultural, religious, economic factors in relation to reproductive health.

This paper presents the case study of HRP’s efforts to build research capacity in Latin America by studying and analyzing the five-year history of institutional development support to an institution in Paraguay. In reviewing the efforts, we identify the strengths in the approaches used by HRP, the challenges and outcomes of the process and we present recommendations for future efforts to strengthen research capacity to improve sexual and reproductive health. The authors call for greater support from and collaborative efforts of developmental partners and governments to strengthen research capacity in low and middle-income countries to improve sexual and reproductive health.

**Keywords:** Research capacity strengthening, institutional development, sexual and reproductive health, low-and middle-income countries (LMICs), grants, policy, practices.
